# Gut metabolome and microbiota signatures predict response to treatment with exclusive enteral nutrition in a prospective study in children with active Crohn’s disease

**DOI:** 10.1016/j.ajcnut.2023.12.027

**Published:** 2024-02-19

**Authors:** Ben Nichols, Anny Briola, Michael Logan, Jaroslav Havlik, Anna Mascellani, Konstantinos Gkikas, Simon Milling, Umer Zeeshan Ijaz, Christopher Quince, Vaios Svolos, Richard K Russell, Richard Hansen, Konstantinos Gerasimidis

**Affiliations:** 1 Human Nutrition, School of Medicine, University of Glasgow, Glasgow Royal Infirmary, Glasgow, United Kingdom; 2 Department of Food Science, Czech University of Life Sciences Prague, Prague, Czech Republic; 3 School of Infection and Inflammation, University of Glasgow, Glasgow, United Kingdom; 4 Civil Engineering, School of Engineering, University of Glasgow, Glasgow, United Kingdom; 5 Organisms and Ecosystems, Earlham Institute, Norwich, United Kingdom; 6 Department of Paediatric Gastroenterology, Hepatology and Nutrition, Royal Hospital for Children and Young People, Edinburgh, United Kingdom; 7 Department of Paediatric Gastroenterology, Hepatology and Nutrition, Royal Hospital for Children, Glasgow, United Kingdom; 8 Department of Child Health, Division of Clinical and Molecular Medicine, School of Medicine, University of Dundee, Dundee, United Kingdom

**Keywords:** exclusive enteral nutrition, Crohn’s disease, microbiome, precision therapy, metabolome, short chain fatty acids, o'link, cytokines

## Abstract

**Background:**

Predicting response to exclusive enteral nutrition (EEN) in active Crohn’s disease (CD) could lead to therapy personalization and pretreatment optimization.

**Objectives:**

This study aimed to explore the ability of pretreatment parameters to predict fecal calprotectin (FCal) levels at EEN completion in a prospective study in children with CD.

**Methods:**

In children with active CD, clinical parameters, dietary intake, cytokines, inflammation-related blood proteomics, and diet-related metabolites, metabolomics and microbiota in feces, were measured before initiation of 8 wk of EEN. Prediction of FCal levels at EEN completion was performed using machine learning. Data are presented with medians (IQR).

**Results:**

Of 37 patients recruited, 15 responded (FCal < 250 μg/g) to EEN (responders) and 22 did not (nonresponders). Clinical and immunological parameters were not associated with response to EEN. Responders had lesser (μmol/g) butyrate [responders: 13.2 (8.63–18.4) compared with nonresponders: 22.3 (12.0–32.0); *P =* 0.03], acetate [responders: 49.9 (46.4–68.4) compared with nonresponders: 70.4 (57.0–95.5); *P =* 0.027], phenylacetate [responders: 0.175 (0.013–0.611) compared with nonresponders: 0.943 (0.438–1.35); *P =* 0.021], and a higher microbiota richness [315 (269–347) compared with nonresponders: 243 (205–297); *P =* 0.015] in feces than nonresponders. Responders consumed (portions/1000 kcal/d) more confectionery products [responders: 0.55 (0.38–0.72) compared with nonresponders: 0.19 (0.01–0.38); *P =* 0.045]. A multicomponent model using fecal parameters, dietary data, and clinical and immunological parameters predicted response to EEN with 78% accuracy (sensitivity: 80%; specificity: 77%; positive predictive value: 71%; negative predictive value: 85%). Higher taxon abundance from *Ruminococcaceae*, *Lachnospiraceae*, and *Bacteroides* and phenylacetate, butyrate, and acetate were the most influential variables in predicting lack of response to EEN.

**Conclusions:**

We identify microbial signals and diet-related metabolites in feces, which could comprise targets for pretreatment optimization and personalized nutritional therapy in pediatric CD.

## Introduction

Treatment with exclusive enteral nutrition (EEN) induces clinical remission in ≤80% of children with active Crohn’s disease (CD), but fewer patients show normalization of gut inflammatory biomarkers, such as fecal calprotectin (FCal) at treatment completion [1,2]. There is currently strong interest in stratified or personalized medicine; particularly for conditions in which response to therapies is variable, such as EEN in CD. The evolution of machine learning and the progress of high-throughput sequencing have begun to answer important questions in the etiology and management of inflammatory bowel disease (IBD), particularly through the integration of multiple parameters such as disease phenotype, blood and immune function markers, and the intestinal microbiota and its metabolites [[3], [4], [5], [6]]. Such technologies and system biology may help predict therapeutic outcomes and lead to a novel understanding of underpinning mechanisms of disease pathogenesis. Integrating this approach into routine clinical care could ultimately allow patient stratification to guide treatment decisions, pretreatment optimization and therefore a more efficacious and cost-effective approach to patient care.

The literature exploring predictive factors of EEN response is sparse and mostly focused on clinical parameters such as disease phenotype [2] and disease severity during the initial period of treatment [7]. Hence, the objective of this study was to analyze, for the first time, to our knowledge, an extensive set of pretreatment factors as predictors of response to EEN. We included disease phenotype and characteristics, anthropometry, dietary intake, routine disease markers, inflammatory cytokines, plasma inflammation-related proteomic markers and diet-related bacterial metabolites, and the metabolome and microbiota in feces; all before EEN initiation. Although widely applied in clinical practice, disease activity indices, such as the weighted pediatric Crohn’s disease activity index (wPCDAI) [8], only very broadly correlate with histological and endoscopic activity [9,10]. We therefore chose FCal, which is more sensitive to detecting endoscopic activity in IBD [9,11] and thus might serve as a more appropriate biomarker for assessing response to EEN in the absence of endoscopy and as a potential “treat-to-target” biomarker.

## Methods

### Subjects

Children with active CD receiving EEN (Modulen IBD) for 8 wk were recruited prospectively at the Royal Hospital for Children, Glasgow, between October 2014 and May 2017. The clinical outcomes of the entire patient cohort were published previously [1]. In the current study, we included only participants who completed EEN and provided paired fecal samples at treatment initiation and completion (Supplemental Table 1 and Supplemental Figure 1). Exclusion criteria included use of antibiotics and probiotics 1 mo prior to EEN initiation, and concomitant use of other induction therapy during EEN. Patients who were administered antibiotics and probiotics during their course of EEN were subsequently excluded too. Clinical and anthropometric parameters, dietary intake, cytokines, inflammation-related blood proteomics, fecal diet-related metabolites, and metabolome and microbiota parameters were explored as predictors of response to treatment with EEN.

### Disease characteristics and clinical parameters

Data on routine blood inflammatory markers [for example, C-reactive protein (CRP), FCal, demographics, anthropometry, wPCDAI, Bristol stool chart score, and disease phenotype] were collected prospectively [12]. FCal was measured in house at the end of the study (Calpro). The primary outcome utilized for prediction of EEN response was normalization of FCal concentration (250 μg/g plus 10% to account for in-house measurement assay inter- and intravariation) at EEN completion.

### Dietary intake

Prior to initiation of EEN, the intake of macronutrients, fiber, and energy was estimated using the Scottish collaborative food frequency questionnaire (FFQ) for children [13]. The 148 food items in the FFQ were grouped under 16 food groups with frequency of portion consumption per day. Macronutrient intake was expressed as a percentage of total energy intake, except for fiber which was expressed as g/1000 kcal/d. Food group intake was standardized as portion/1000 kcal/d.

### Plasma inflammatory cytokines and inflammation-related proteomics

The absolute concentration of 19 cytokines and relative concentration of 92 inflammation-related proteomic markers were measured in plasma with the Meso Scale Diagnostics platform (Meso Scale Diagnostics) and Olink assays (Olink Proteomics), respectively.

### Diet-related bacterial metabolites and fecal metabolomics

The entire bowel motion was collected fresh in an empty, disposable container. Immediately following defecation an anaerobic sachet (Anaerocult A, Merck) was placed above the recipient container to reduce oxygen concentration and the samples were placed in a cool bag along with icepacks. Within 4 h of defecation, samples were transported to the laboratory, homogenized using mechanical kneading with a blender and aliquots were stored for downstream analyses. Aliquots for the measurement of short-chain fatty acids (SCFAs, stabilized with NaOH 1M) and total (stabilized with zinc acetate 0.11M) and free sulfide (stabilized with NaOH 1.25M) were stored in −20°C, whereas aliquots intended for LC-MS, Proton nuclear magnetic resonance (^1^H NMR) metabolomics and microbiota analysis were stored in −80°C. In feces, the concentration of short (SCFA), branched, and medium chain fatty acids was measured using gas chromatography coupled with a flame ionization detector (GC) (Supplemental Methods), and ammonia was measured on the day of sample collection with an automated analyzer (HI 96715, Hanna Instruments), and free/total sulfide in stored samples with colorimetric assays [14]. Fecal pH and fecal water content (%) were also measured [15]. ^1^H NMR metabolomics of fecal samples was performed using a 500 MHz spectrometer, using a One dimension Nuclear Overhauser Effect Spectroscopy pulse sequence with water suppression (Supplemental Methods). Comparisons were carried out in annotated and quantified metabolites [16].

### Fecal microbiota

Genomic DNA was extracted from feces within 2 mo of sample collection, as described previously and in Supplemental Methods [17]. Total bacterial load in feces was measured by qPCR (forward primer: CGG TGA ATA CGT TCC CGG and reverse primer: TAC GGC TAC CTT GTT ACG ACT T) using Taqman chemistry [14]. The V4 region of the 16S rRNA gene was sequenced (MiSeq) in fecal samples using 2 × 250-bp paired-end reads. The V4 region was amplified (forward primer: GTGCCAGCMGCCGCGGTAA and reverse primer: GGACTACHVGGGTWTCTAAT) using fusion Golay adapters barcoded on the reverse strand. Barcoded amplicons were purified using the Zymoclean Gel DNA Recovery Kit (D4001, Zymo Research).

### Bioinformatics

Operational taxonomic units (OTUs) were constructed from the raw 16S rRNA sequencing data at a similarity of 97% using the VSEARCH pipeline (https://github.com/torognes/vsearch/wiki/VSEARCH-pipeline) [18]. Paired reads were merged and quality filtering was performed with a maximum expected error value of 0.5. Sequences longer than 275 bp and shorter than 225 bp were discarded, reads were dereplicated across all samples and singleton sequences were filtered out. Chimeras were identified and eliminated using the VSEARCH implementation of the UCHIME de novo algorithm after preclustering at 98%. The UCHIME reference-based chimera detection method was then applied using the “Gold” ChimeraSlayer database [19]. OTUs were generated by clustering the remaining sequences at 97%. Taxonomic classification to genus level was performed using the Ribosomal Database Project Naïve Bayes Classifier algorithm in conjunction with the SILVA (version 123) database [20,21].

In plasma proteomic and fecal metabolome analysis, data below the limit of detection were replaced by half of the minimum detected value. For multivariate analysis, the fecal metabolite concentrations from ^1^H NMR analysis initially expressed as μg/g of wet fecal sample, were further normalized by total sum, log-transformed, and scaled using unit variance scaling, whereby the data are mean-centered and divided by the standard deviation for each variable.

### Statistical analysis

Statistical analyses were carried out with Excel (version 2310, Microsoft Corporation) and R statistical software (version 4.2.3, R Foundation) with RStudio (version 2023.03.0, Posit Software). For nonparametric data, Mann–Whitney U tests were used for all comparisons between groups, and Spearman’s rank correlation test was performed for correlations. Continuous variables were summarized with medians and IQR, and categorical parameters were presented by counts and frequencies.

Fecal microbial community structure was analyzed using the vegan R package, and responders and nonresponders were compared in terms of α diversity, using the Chao1 richness estimate and Shannon diversity index, and β diversity, using Bray–Curtis dissimilarity, nonmetric multidimensional scaling and β dispersion [22]. For taxon abundance analysis, we normalized the dataset using total sum scaling normalization combined with centered log-ratio transformation. We removed low abundant features by keeping taxa that accounted for >0.01% of all reads. All microbial diversity and taxon abundance analyses were carried out at the OTU level.

The plasma inflammation-related proteomic profile was evaluated using the performance of principal components analysis (PCA). Separation between groups was assessed with the use of permutation analysis of variance (ANOVA) tests on Euclidean distance matrices. Discriminant proteins were identified with the use of Mann–Whitney U tests. Results of differential analysis for all datasets were corrected for multiple testing with the Benjamini–Hochberg method. *P* values below 0.05 were considered statistically significant. For multivariate analysis of the ^1^H NMR metabolome data, PCA and Orthogonal Projections to Latent Structures Discriminant Analysis ordination were applied to identify differences between groups using MetaboAnalystR. Levels of individual metabolites were compared using Mann–Whitney tests between groups.

### Random forest modeling

For the prediction of EEN response from the various datasets, we generated random forest (RF) models, which use a machine learning algorithm widely applied for classification and prediction purposes on multiomics data [23]. RF analysis was performed using the R package, randomForest [24], separately for microbiota, ^1^H NMR metabolome, cytokines, inflammation-related proteomics, SCFA, dietary intake, routine clinical datasets, and inflammatory biomarkers (for example, CRP and FCal) at EEN initiation. Variable optimization was applied using the FeatureTerminatoR R package which minimizes the number of variables in the model without reducing model performance. For all models, 50,000 decision trees were grown and candidate variables at each split were set to default. To account for class imbalances, the data were stratified by response type. The importance of each feature in the model was assessed as mean decrease in Gini impurity index, which shows the change in classification accuracy between a model with and without the variable of interest, with the Gini impurity representing the probability that a specific sample will be classified incorrectly when labeled randomly. Model significance was determined after running a permutation test 1000 times using the rf.significance function in the rfUtilities R package [25]. Finally, a receiver operating characteristic (ROC) curve was plotted, and the AUC was calculated with the R package pROC. In multicomponent analysis, all datasets were combined in a single RF model. In multicomponent analysis, missing data were replaced by the corresponding variable medians.

### Ethics

The study was approved by the West of Scotland Research Ethics Committee (14/WS/1004) and registered at clinicaltrials.gov (NCT02341248). All patients/carers provided informed consent. All authors had access to the study data, and reviewed and approved the final manuscript.

## Results

### Participant characteristics and clinical parameters

Of the 66 children with active CD, 54 completed EEN (Supplemental Figure 1). For 37 of 54 (69%) patients, FCal was measured at both baseline and EEN completion and these children were included in the current study. At EEN initiation, all patients had FCal levels of >250 μg/g. Of these, 15 of 37 (41%) displayed FCal levels of ≤250 μg/g after EEN and were classified as responders. The rest of the patients with FCal levels of >250 μg/g were classified as nonresponders (*n* = 22/37, 59%). There were no differences in disease characteristics between patients included in this study and those in the complete cohort (Supplemental Table 1). Pretreatment wPCDAI, FCal, anthropometry, disease phenotype, use of immunosuppressants, and routine inflammatory biomarkers in blood were not different between the 15 responders and the 22 nonresponders (Table 1). An RF using participants’ characteristics and clinical parameters failed to differentiate between responders and nonresponders (permutation test *P =* 0.158).

**TABLE 1:** Pretreatment patient characteristics in responders and nonresponders to treatment with exclusive enteral nutrition

Variables^1^	Responders (*N* = 15)	Nonresponders (*N* = 22)	*P* value
Age (y)	12.1 (10.6, 14.8)	12.4 (10.3, 14.8)	0.99
Sex, male, *n* (%)	11 (73)	14 (64)	0.36
BMI *z*-score	−0.74 (−1.73, −0.06)	−0.23 (−1.19, 0.32)	0.15
Height *z*-score	−0.43 (−0.96, 0.27)	−0.06 (−0.53, 0.62)	0.31
Weight *z*-score	−0.85 (−1.52, −0.32)	−0.30 (−0.88, 0.08)	0.18
wPCDAI	27.5 (16.3, 46.3)	41.3 (27.5, 57.5)	0.15
wPCDAI classification, *n* (%)			0.84
Severe (wPCDAI > 57.5)	3 (20)	5 (23)	
Moderate (wPCDAI > 40)	3 (20)	6 (27)	
Mild (wPCDAI > 12.5)	9 (60)	10 (45)	
Remission (wPCDAI ≤ 12.5)	0 (0)	1 (5)	
FCal (μg/g) (*n =* 34)	1352 (863, 1857)	1699 (1386, 1835)	0.17
CRP (mg/L) (*n =* 34)	5.00 (2.25, 20.5)	14.0 (3.00, 25.5)	0.49
Albumin (g/L) (*n =* 36)	34.5 (26.8, 38.0)	32.5 (27.0, 37.0)	0.99
ESR (mm/h) (*n =* 30)	12 (8.5, 32.5)	19 (13, 35.5)	0.49
Hemoglobin (g/L) (*n =* 34)	126 (124, 128)	119 (111, 130)	0.39
Use of immunosuppressants, *n* (%)	7 (46.7)	10 (45.5)	0.94
Azathioprine, *n* (%)	7 (46.7)	8 (36.4)	
Mercaptopurine, *n* (%)	0 (0)	1 (4.55)	
Methotrexate, *n* (%)	0 (0)	1 (4.55)	
Disease location, *n* (%)			0.33
L1	1 (7)	2 (9)	
L2	2 (13)	3 (14)	
L2, L4a	1 (7)	4 (18)	
L2, L4a, L4b	1 (7)	1 (5)	
L3	3 (20)	3 (14)	
L3, L4a	0 (0)	7 (32)	
L3, L4b	1 (7)	0 (0)	
L3, L4a, L4b	6 (40)	2 (9)	
Perianal involvement, *n* (%)	2 (13)	2 (9)	1.00
FCal post-EEN (μg/g)	133 (37, 244)	1047 (735, 1705)	<0.001
wPCDAI post-EEN	0 (0, 7.5)	7.5 (0, 19.4)	0.07

Abbreviations: CRP, C-reactive protein; EEN, exclusive enteral nutrition; ESR, erythrocyte sedimentation rate; FCal, fecal calprotectin; L1, distal 1/3 ileal ± limited caecal disease; L2, colonic; L3, ileocolonic; L4a, upper disease proximal to Ligament of Treitz; L4b, upper disease distal to Ligament of Treitz and proximal to distal 1/3 ileum; wPCDAI, weighted pediatric Crohn’s disease activity index.

1 Data are presented as median (IQR) or numbers. Chi-square tests were used for categorical variables, and Mann–Whitney U tests for numerical variables.

### Pretreatment dietary intake in responders and nonresponders

**FIGURE 1:**
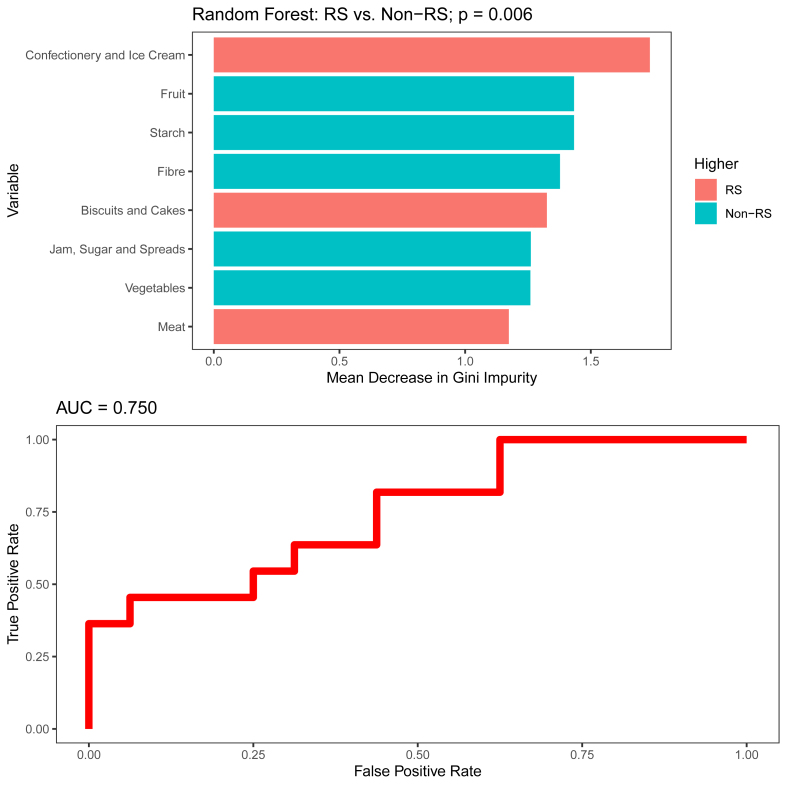
Random forest classification between responders and nonresponders using the intakes of macronutrients, fiber, and food groups from food frequency questionnaires. Starch was reported as percentage energy intake and the other variables per 1000 kcal/d. The top 15 variables with the highest mean decrease in Gini impurity and ROC curve are shown. Non-RS, fecal calprotectin nonresponders; ROC, receiver operating characteristic; RS, fecal calprotectin responder.

Treatment responders reported a higher baseline intake of confectionery and ice cream products compared with nonresponders [median (IQR), responders: 0.55 portions/1000 kcal/d (Q1: 0.38, Q3: 0.72) compared with nonresponders: 0.19 portions/1000 kcal/d (Q1: 0.01, Q3: 0.38); *P =* 0.045] (Table 2). No other significant differences were observed in macronutrient or food group intake between the 2 groups, except for a marginally significant (*P =* 0.09) higher fiber intake in nonresponders (Table 2). An RF model, using the intakes of food groups (portions/1000 kcal) and macronutrients (% of energy intake) as input variables, yielded an accuracy of 67% with sensitivity of 55%, specificity of 75%, positive predictive validity (PPV) of 60%, and negative predictive validity (NPV) of 71%, (permutation test *P =* 0.006) to predict response (Figure 1). The foods that contributed the most to the model were confectionery, which were higher in responders, and fruit, starch, and fiber, which were all higher in nonresponders.

**TABLE 2:** Pretreatment dietary intake in responders and nonresponders to treatment with exclusive enteral nutrition

Variables^1^	Responders (*N* = 11)	Nonresponders (*N* = 16)	*P* value
Energy and nutrient intake			
Energy intake (kcal)	1322 (1213, 1658)	1525 (1365, 1712)	0.42
%EAR	66.0 (50.3, 84.6)	64.7 (52.9, 81.2)	0.86
Protein (g)	49.7 (42.7, 57.7)	53.5 (44.7, 65.9)	0.61
% energy from protein	13.8 (12.6, 16.2)	13.6 (12.8, 14.7)	0.67
Fat (g)	52.4 (46.4, 63.1)	55.3 (44.1, 58.0)	0.98
% energy from fat	34.6 (32.0, 35.7)	33.3 (30.0, 35.5)	0.36
Saturated fatty acids (g)	21.5 (18.7, 27.1)	22.0 (15.4, 25.9)	0.90
% energy from saturated fatty acids	14.5 (13.2, 16.0)	13.6 (12.4, 15.1)	0.15
Carbohydrates (g)	180 (156, 222)	215 (188, 258)	0.37
% energy from carbohydrates	54 (51.7, 55.9)	55.3 (53.2, 61.0)	0.23
Sugars (g)	77.1 (58.4, 96.2)	85.9 (66.1, 107)	0.68
% energy from sugars	24.9 (20.7, 26.7)	22.0 (20.6, 26.4)	0.61
Fiber (g)	8.7 (6.94, 10.1)	10.7 (8.76, 12.4)	0.13
Fiber/1000 kcal	6.37 (5.8, 6.78)	7.03 (6.18, 7.92)	0.09
Starch (g)	96 (85.5, 114.5)	113 (97.8, 142)	0.19
% energy from starch	27.8 (26.0, 29.3)	32.4 (26.1, 36.2)	0.16
Food groups (portions per day per 1000 kcal)			
Breakfast cereals	0.823 (0.683, 1.189)	1.13 (0.356, 1.775)	0.35
Bread	0.994 (0.404, 1.381)	1.073 (0.517, 1.669)	0.68
Milk	0.85 (0.537, 1.341)	1.242 (0.637, 1.56)	0.51
Yogurt, cheese, and eggs	0.458 (0.278, 0.833)	0.342 (0.201, 0.579)	0.37
Meat	1.033 (0.883, 1.352)	0.822 (0.558, 1.369)	0.25
Fish	0.138 (0.081, 0.278)	0.215 (0.112, 0.353)	0.43
Potatoes, rice, and pasta	0.807 (0.631, 1.012)	0.885 (0.752, 1.129)	0.61
Savory dishes	1.05 (0.535, 1.147)	0.738 (0.497, 1.076)	0.65
Vegetables	0.486 (0.283, 0.955)	1.052 (0.312, 1.445)	0.27
Fruit	0.425 (0.334, 0.732)	0.768 (0.557, 1.07)	0.15
Juices and drinks	3.649 (2.407, 3.942)	2.446 (1.704, 4.218)	0.65
Jam, sugar, and spreads	0.137 (0, 0.428)	0.496 (0.046, 1.287)	0.16
Crisps, nuts, and savory snacks	0.496 (0.396, 0.696)	0.447 (0.281, 0.672)	0.61
Biscuits and cakes	1.216 (0.652, 1.366)	0.497 (0.3, 1.02)	0.11
Desserts	0.027 (0, 0.201)	0.028 (0, 0.109)	0.86
Confectionery and ice cream	0.55 (0.38, 0.72)	0.19 (0.01, 0.38)	0.045

Abbreviation: EAR, estimated average requirements.

1 Data are presented as median (IQR). Mann–Whitney U tests were used for statistical analysis.

### Pretreatment diet-related bacterial metabolites in responders and nonresponders

Pretreatment concentration (μmol/g) of fecal butyrate, measured with GC, in responders was about half that of nonresponders, when data expressed per dry [median (IQR), responders: 64.9 (Q1: 48.0, Q3: 86) compared with nonresponders: 112 (Q1: 65.7, Q3: 162); *P =* 0.04] or per wet fecal matter [median (IQR), responders: 13.0 (Q1: 7.79, Q3: 16.9) compared with nonresponders: 20.6 (Q1: 7.99, Q3: 30.5); *P =* 0.06] (Supplemental Table 2). This signal was not an artifact of differences in microbial load (*P =* 0.12), or fecal water content between the 2 groups, as neither fecal water content (*P =* 0.87) nor Bristol stool scale (*P =* 0.83) differed between the 2 groups (Supplemental Table 2). A similar trend (*P =* 0.09) was observed for acetate (Supplemental Table 2). Fiber intake, from dietary analysis, correlated positively with acetate (dry matter: rho = 0.50; *P =* 0.017 and wet matter: rho = 0.52; *P =* 0.013) but not with propionate or butyrate. No significant differences were observed in feces between the 2 groups in pH, ammonia, and free or total sulfide (Supplemental Table 2). An RF using diet-related metabolites in feces failed to predict response to EEN (permutation test *P =* 0.144).

### Pretreatment fecal ^1^H NMR metabolome in responders and nonresponders

**FIGURE 2:**
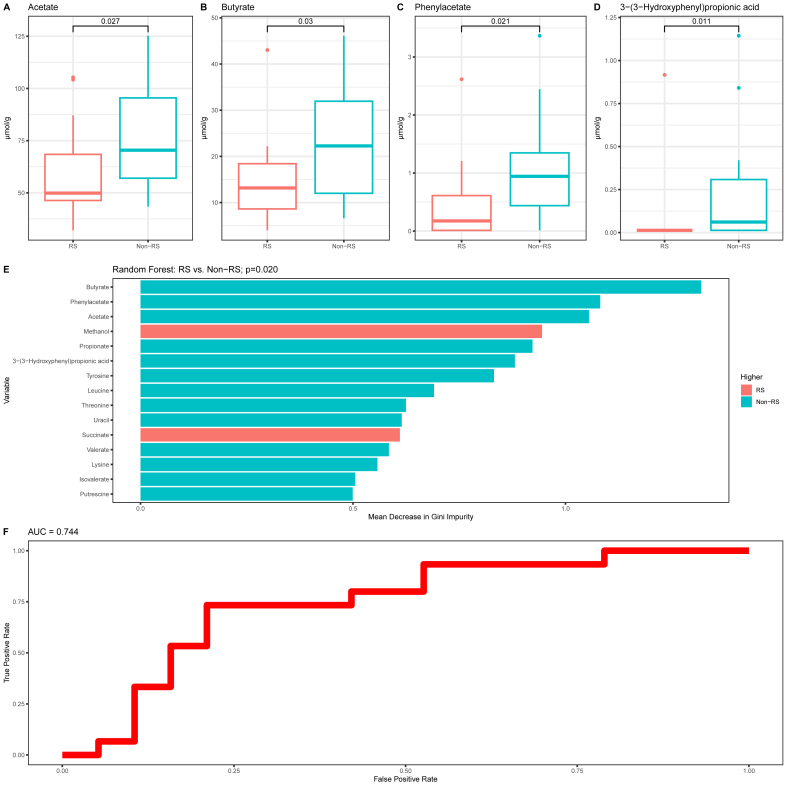
(A–D) Pretreatment discriminant ^1^H NMR fecal metabolites between responders and nonresponders with mean decrease in (E) Gini impurity and (F) ROC curve for random forest classification. ^1^H NMR, proton nuclear magnetic resonance; non-RS, fecal calprotectin nonresponder; ROC, receiver operating characteristic; RS, fecal calprotectin responder.

Twenty-nine metabolites from ^1^H NMR analysis were annotated (Supplemental Table 3). No difference in global metabolome structure was found (permutation ANOVA *P =* 0.737; Supplemental Figure 2), but significant differences were discovered for individual metabolites and, in accordance with findings observed for the same metabolites quantified with GC (Supplemental Table 2). Thus, the pretreatment concentration of fecal butyrate (μmol/g) was significantly lower in responders than nonresponders [median (IQR), responders: 13.2 (Q1: 8.63, Q3: 18.4) compared with nonresponders: 22.3 (Q1: 12.0, Q3: 32.0); *P =* 0.03], and so was the case for the concentration of acetate [median (IQR), responders: 49.9 (Q1: 46.4, Q3: 68.4) compared with nonresponders: 70.4 (Q1: 57.0, Q3: 95.5); *P =* 0.027], phenylacetate [median (IQR), responders: 0.175 (Q1: 0.013, Q3: 0.611) compared with nonresponders: 0.943 (Q1: 0.438, Q3: 1.35); *P =* 0.021] and 3-(3-hydroxyphenyl)propionic acid [median (IQR), responders: 0.013 (Q1: 0.013, Q3: 0.013) compared with nonresponders: 0.061 (Q1: 0.013, Q3: 0.308); *P =* 0.011] (Figure 2A–D). An RF model, using the levels of all ^1^H NMR metabolites as input, produced an accuracy of 74% with sensitivity of 73%, specificity of 74%, PPV of 69% and NPV of 78% (permutation test *P =* 0.02) to predict response to EEN. The most influential metabolites in this model were butyrate followed by phenylacetate and acetate with concentrations much higher in nonresponders (Figure 2E, F). Acetate correlated strongly with butyrate (*r* = 0.72; *P <* 0.001) and both SCFA with 3-(3-hydroxyphenyl)propionic acid (acetate [*r* = 0.46; *P =* 0.007]; butyrate [*r* = 0.45; *P =* 0.008]). No correlations were observed between these 3 metabolites with phenylacetate, suggesting a dependent relationship between the 3 former metabolites, but none between them and phenylacetate.

### Pretreatment inflammation-related plasma proteome and cytokines in responders and nonresponders

The absolute concentration of 19 inflammatory cytokines measured with the multiplex assay did not differ between responders and nonresponders (Supplemental Table 4). There was no clustering of inflammation-related proteome structure according to the EEN response (permutation ANOVA *P =* 0.737) (Supplemental Figure 3) and an RF using those 19 inflammatory cytokines did not predict EEN (permutation test *P =* 0.293). From the 92 inflammation-related proteomic markers measured with the Olink technology, the concentration of NT-3 was lower in responders [median (IQR), responders: 0.69 (Q1: 0.69, Q3: 0.69) compared with nonresponders: 1.49 (Q1: 0.69, Q3: 1.79); *P =* 0.03], whereas the concentration of CXCL6 was higher in responders [median (IQR), responders: 11.1 (Q1: 10.5, Q3: 11.2) compared with nonresponders: 10.5 (Q1: 10.3, Q3: 10.9); *P =* 0.046] (Supplemental Table 5). However, an RF generated with the 92 proteomic markers was not predictive of response to EEN (permutation test *P =* 0.473).

### Pretreatment fecal microbiota in responders and nonresponders

**FIGURE 3:**
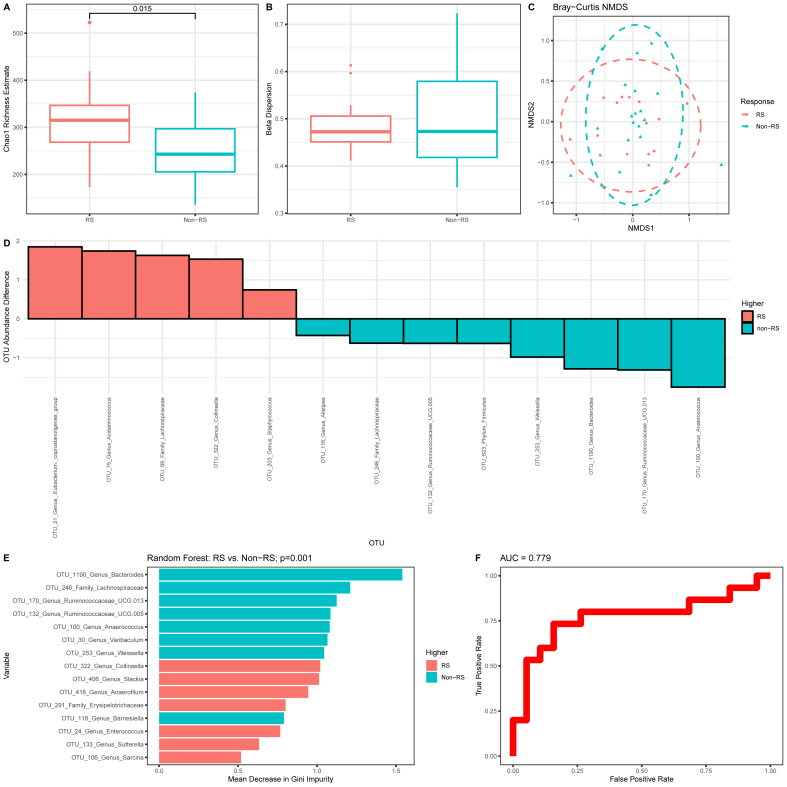
Pretreatment fecal microbiome characteristics in responders and nonresponders. (A) Chao1 richness estimate, (B) β dispersion, (C) Bray–Curtis NMDS ordination, and (D) significantly discriminant OTUs. The top 15 OTUs with the highest mean decrease in (E) Gini impurity and (F) ROC curve are shown for random forest classification. NMDS, nonmetric multidimensional scaling; non-RS, fecal calprotectin nonresponder; OUT, operational taxonomic unit; ROC, receiver operating characteristic; RS, fecal calprotectin responder.

16S sequencing yielded a mean of 49,238 reads per sample (minimum: 10,039; maximum: 106,518; SD: 29,300). Microbiota Chao1 richness (α diversity) was higher in responders [median (IQR), responders: 315 (Q1: 269, Q3: 347) compared with nonresponders: 243 (Q1: 205, Q3: 297); *P =* 0.015] (Figure 3A) but community composition (β diversity) did not differ significantly between the 2 groups (Figure 3B, C). Of the 238 OTUs, 13 OTUs were differentially abundant between groups (Figure 3D). RF analysis produced a model with an accuracy of 79%, sensitivity of 73%, specificity of 84%, PPV of 79%, and NPV of 80% (permutation test *P =* 0.001) to differentiate between responders and nonresponders (Figure 3E, F). The most influential OTUs, which all had a higher abundance in nonresponders than responders, were assigned to *Bacteroides*, *Lachnospiraceae*, *Ruminococcaceae*, and *Anaerococcus* (Figure 3E).

### Multicomponent prediction of responses to EEN

**FIGURE 4:**
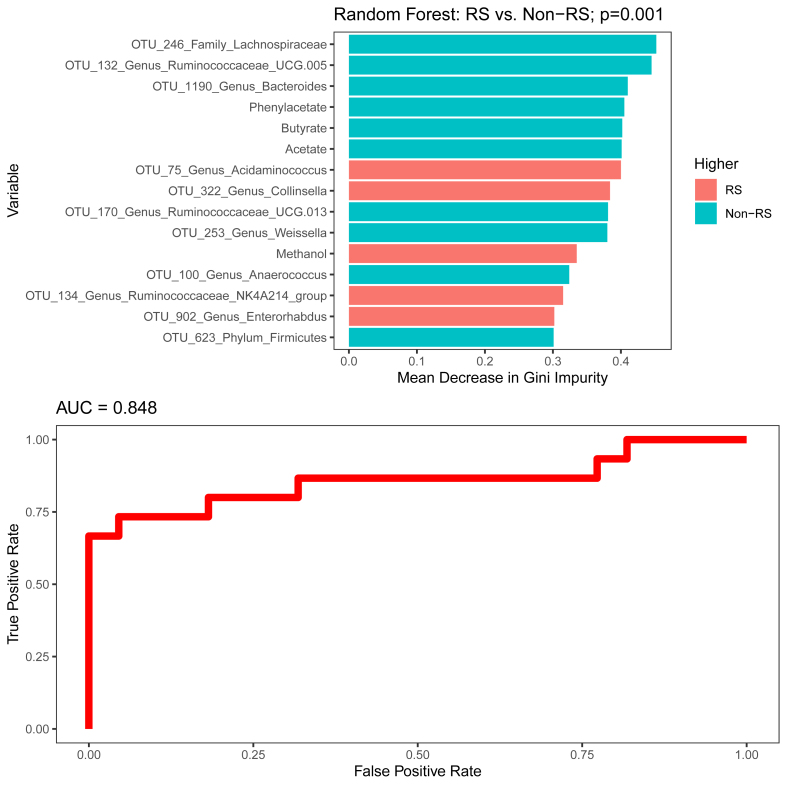
Random forest classification between responders and nonresponders using a multicomponent model with fecal OTUs, ^1^H NMR metabolites, Chao1 richness estimate of the fecal microbiome, cytokine, proteomic, diet-related metabolites, dietary intake, disease, and routine clinical datasets. The top 15 variables with the highest mean decrease in Gini impurity and ROC curve are shown. ^1^H NMR, proton nuclear magnetic resonance; non-RS, fecal calprotectin nonresponder; OUT, operational taxonomic unit; ROC, receiver operating characteristic; RS, fecal calprotectin responder.

Last, a multicomponent RF model was generated by including the entire study’s clinical parameters and omics datasets in the model, except for SCFA measured with GC to avoid replication of the same data measured with ^1^H NMR. The final model yielded an accuracy of 78%, sensitivity of 80%, specificity of 77%, PPV of 71%, and NPV of 85% (permutation test *P =* 0.001) (Figure 4).

The most influential variables in this model were OTUs from *Lachnospiraceae, Ruminococcaceae*, and *Bacteroides,* phenylacetate, butyrate, and acetate, all of which were higher in nonresponders. Conversely, OTUs from *Acidaminococcus and Collinsella*, which were higher in responders, were the most influential variables of response to EEN. The concentration of the 3 differential metabolites (that is, butyrate, acetate, and phenylacetate) correlated significantly with the relative abundances of several differential OTUs from the same model and, most importantly, retained the direction of their effect in predicting responses to EEN (Supplemental Figure 4).

## Discussion

The present study found that pretreatment dietary, microbiota and metabolomic gut signatures can predict with high accuracy 8 of the 10 patients who will show an FCal response to EEN; thus, closing the gap routine clinical parameters were unable to fill. Pretreatment butyrate, acetate, and phenylacetate concentrations were at higher levels in nonresponders; almost double those of responders. These findings are counterintuitive, because butyrate is associated with anti-inflammatory pathways in intestinal mucosa [26], and the levels of several butyrate-producing species have been consistently reported to be reduced in CD [27]. It is unlikely the increased pretreatment levels of fecal acetate and butyrate in nonresponders stem from diminished absorption because of more extensive inflammation in colonocytes, as the same strength of association was not observed across all SCFA measured and neither FCal levels nor wPCDAI differed between the 2 study groups. Previous research found a decrease in fecal butyrate during EEN [14,15], but it remains unclear whether this is simply an epiphenomenon of the lack of fiber in EEN feed composition [28], or if it is causally involved in its mechanisms of action [29]. In a recent study, fecal butyrate levels associated positively with higher FCal levels during early food reintroduction post-EEN [30] and another research group showed that unfermented β fructans exacerbated inflammation in certain patients with active IBD [31]. This unexpected positive relationship between fiber intake, SCFA production, at treatment initiation, and colonic inflammation at completion of EEN requires further investigation.

Phenylacetate was also a strong predictor of FCal responses to EEN, both in single and multicomponent models. Phenylacetate is a catabolic product of phenylalanine and other aromatic compounds which is further metabolized by selected species including *Escherichia coli*. Its exact role in CD has not been described but it might comprise a biomarker of metabolism of certain pathobionts, such as adherent and invasive *E. coli*, which have been implicated extensively in the pathogenesis of CD [32]. The fact that only diet-related bacterial metabolites and organisms which are major fiber fermenters in the gut differentiated between responders and nonresponders, in the current study, further underlines the importance of dietary factors in the management of active CD and possibly its underlying etiology.

Patients with higher microbiota diversity in feces benefited the most from EEN. Reduced bacterial richness is a consistent finding in fecal and mucosal samples of patients with CD, compared with healthy controls [27] and has been correlated with the extent of intestinal inflammation, assessed using FCal, in pediatric CD [33]. In contrast, no differences in α diversity metrics were observed between patients who had a >50% decrease in FCal levels and others who did not, in another study [34] suggesting that specific organisms may be more important than crude metrics of community structure. Indeed, in multicomponent analysis using machine learning, the influence of microbiota richness in predicting response to EEN became less important and certain bacterial taxa became more important predictors.

Although microbial signals identified could have prognostic value to screen patients whose gut inflammation will improve with EEN, and therefore personalize treatment options, their role in the primary disease pathogenesis is difficult to decipher within the current study. Randomized controlled trials can address such mechanistic questions where FCal responses to EEN will be measured after dietary or pharmacological manipulation of prognostic microbial species and metabolites identified in the present study, prior to EEN initiation*.* Based on the findings of this present study a potential intervention might be a diet low in precursors of phenylacetate production, such as a diet low in phenylalanine or protein. Nonetheless, common species previously associated with the pathogenesis of CD [27], such as *Akkermansia muciniphila*, *E. coli*, and *Veillonella*, were not identified as predictors of EEN response in this study. It is also possible that we have observed different subsets of patients with different underlying microbial origins of CD, despite their similar immunity and disease phenotype. One subset of patients in which disease is driven by *E. coli* pathobionts which produce phenylacetate and in which EEN works by reducing their abundance, and another subset of patients where disease is driven by other butyrate-producing microbes such as *Ruminococcaceae*, for which EEN works by depleting fiber substrate they require for growth.

The main limitations of the current study include small sample size meaning that for some analyses statistical power may have been low, particularly when the original study was designed to test different primary outcomes, plus the lack of independent replication in larger studies. The results of these studies may also be relevant only to the local Scottish population of children with CD, treated for 8 wk with Modulen IBD. Hence, replication of the current findings in a cohort of patients of different ethnic background and with use of other EEN feeds may be required before generalization of study findings can be made.

Using a multiomics approach, we identified pretreatment microbial species and diet-related metabolites associated with improvement in colonic inflammation during EEN. Should these microbial signals be replicated in independent multicenter research, this would open opportunities for personalized nutritional therapy in CD. Important diet-related metabolites identified here can be measured quickly, noninvasively, and can be further modified with dietary, pharmacological, or other microbiome-modifying treatments prior to an EEN course.

## Author contributions

The authors’ responsibilities were as follows – KG, RKR, RH: conceptualized the study; ML, VS, KGk, JH, SM: carried out patient recruitment and performed laboratory analysis; BN, AB, AM, JH, UZI, CQ: performed bioinformatics and statistical analysis; KG, RKR: supervised the project; KG, BN, AB: produced the first draft; and all authors: read and approved the final manuscript.

## Conflict of interest

RKR reports speaker’s fees, travel support, and advisory boards: Nestle, AbbVie, Celltrion & Pharmacosmos. KG reports personal fees from Nutricia, research grants, and personal fees from Nestle Health Science, and personal fees from Dr Falk Pharma, Abbott, Servier, Mylan, and Baxter. The rest of the authors have no conflicts of interest to declare.

## Funding

This research received funding from The Glasgow Children’s Hospital Charity, Nestle Health Science and The Leona M. and Harry B. Helmsley Charitable Trust. ML received a studentship from the Engineering and Physical Sciences Research Council (EPSRC) and the Nestle Health Science. UZI was funded by the Natural Environment Research Council (NERC NE/L011956/1) and supported by the EPSRC (EP/P029329/1 and EP/V030515/1). BN was partially funded by the Biotechnology and Biological Sciences Research Council (BB/R006539/1).

## Data availability

Anonymized data may become available to third parties after request to the corresponding author and for only for those patients who provided written consent for this specific aspect of study participation.
