# Supplementary material for: Gut metabolome and microbiota signatures predict response to treatment with exclusive enteral nutrition in a prospective study in children with active Crohn’s disease
Source: Am J Clin Nutr. 2024 Feb 19;119(4):885–95. doi: 10.1016/j.ajcnut.2023.12.027 (PMC11007740; doi:10.1016/j.ajcnut.2023.12.027)
Supplement: Multimedia component 2 [file mmc2.docx]

Ben Nichols. “Gut metabolome and microbiota signatures predict response to treatment with exclusive enteral nutrition in a prospective study in children with active Crohn’s disease”

**DNA extraction from fecal samples**

In brief, 50 mg of freeze-dried feces were suspended in a buffer containing guanidine thiocyanate and N-lauroylsarcosine and incubated at 70 °C. Bacterial cells were lysed using silica beads (0.1 mm, Biospec products) in a FastPrep-24 bead beater (MP biomedicals). Subsequently, polyvinylpyrrolidone were added, and the supernatant was retrieved after centrifugation. After three washes with TENP buffer (Tris-Cl, EDTA, NaCl and polyvinylpyrrolidone), and additional centrifugation, the supernatants were pooled and ice-cold isopropanol was added to facilitate the precipitation of nucleic acids. After incubation at room temperature and centrifugation, the supernatant was discarded. The resulting pellet was resuspended in phosphate buffer and potassium acetate and was left at 4 °C overnight. RNAse and Proteinase K were added and after incubation, DNA precipitation was achieved using sodium acetate and ice-cold 100% ethanol. After incubating at −20 °C for 1 hour, the DNA pellet underwent three washes with 70% ethanol, was dried, and stored at −20 °C in TE buffer. All chemicals were purchased from Merck unless otherwise stated. The full protocol can be made available on request by the corresponding author.

**Measurement of faecal SCFA using Gas Chromatohraphy**

The levels of SCFA (acetic acid, propionic acid, butyric acid, valeric acid, caproic acid, heptanoic acid, and caprylic acid) and branched SCFA (isobutyric acid and isovaleric acid) in faecal samples were quantified. Briefly, the SCFA and branched SCFA (isobutyric acid and isovaleric acid) were extracted from 100 mg of feeze dried faeces, acidified with orthophosporic acid, and extracted three times in total using diethyl ether. Extracts were analyzed using gas chromatography (Agilent 7890A) with flame ionization detector (250°C) and Zebron ZB-Wax capillary column (15 m x 0.53 mm x 1 μm) made of polyethylene glycol (Phenomenex, Cheshire, UK). The carrier gas was Nitrogen (30 ml/min). One microlitre of ether extract was automatically injected (230°C, splitless) into the column. The column temperature was held at 80°C for 1 min, increased at 15°C/min until 210°C and held for 1 min. The chromatograms were analysed using OpenLab CDS ChemStation (Agilent, UK).

**Measurement of untargeted faecal metabolites using ^1^H NMR analysis**

Stool samples were diluted in PBS buffer 200 mg/mL, sonicated for 5 min, vortexed and centrifuged at 24,400 ×*g* for 15 minutes at 4°C. To each 630 µL of the resulting supernatant was added 70 µL of sodium phosphate buffer pH 7.4 (1.5M K_2_HPO_4_ + 1.5M Na_2_HPO_4_) in deuterium oxide including 5 mM 3-(trimethylsilyl) propionic-2,2,3,3-*d*4 acid sodium salt (99%, TSP) and 0.2% NaN_3._ The mixture was than centrifuged at 24,400 ×*g* for 15 min at 4°C. Finally, 600 µL of the resulting supernatant were transferred to a 5 mm standard NMR tube pending ^1^H NMR measurements. All chemicals and reagents used were of analytical grade. All ^1^H NMR spectra were recorded using a 500 MHz Bruker AVANCE III HD spectrometer equipped with a broad band fluorine observation (BBFO) SmartProbe^TM^ with z-axis gradients (Bruker, Rheinstetten, DE). Spectral acquisition was performed at 298K using a Bruker pulse sequence *noesypr1d* (recycle delay-90º-t1-90º-tm-90º-acquisition), with delay time of 1 s and mixing time of 100 ms. Spectra were acquired under an automation procedure (tuning, matching, locking, shimming and sample loading) with 192 scans after 4 dummy scans into 64k data points with a spectral width of 16 ppm. The free induction decays obtained were multiplied by a 0.3 Hz line-broadening factor prior to Fourier transformation. Calibration to the internal standard (TSP, 0 ppm), 0.3 Hz apodisation, phase correction, baseline correction and alignment were carried out using Mnova software, version 14.1.0 (Mestrelab Research, Santiago de Compostela, ES). Quantification of metabolites were carried out on the processed spectra by Chenomx NMR suite 8.4 software (Chenomx, Edmonton, CA) using the inbuild reference library version 10 and an in-house build library. The concentrations were normalised to wet weight of each sample.
