# Supplementary material for: Gut metabolome and microbiota signatures predict response to treatment with exclusive enteral nutrition in a prospective study in children with active Crohn’s disease
Source: Am J Clin Nutr. 2024 Feb 19;119(4):885–95. doi: 10.1016/j.ajcnut.2023.12.027 (PMC11007740; doi:10.1016/j.ajcnut.2023.12.027)
Supplement: Multimedia component 3 [file mmc3.docx]

Ben Nichols. “Gut metabolome and microbiota signatures predict response to treatment with exclusive enteral nutrition in a prospective study in children with active Crohn’s disease”

**Supplementary Table 1:** Baseline demographic, anthropometric, clinical, and phenotypic characteristics of the main cohort and patient subset used in the current study^1^.

| Variables ^1^ | Main cohort (N=66) | Current study (N=37) |
| --- | --- | --- |
| Sex, male n (%) | 42 (64) | 25 (68) |
| Newly Diagnosed, n (%) | 60 (91) | 34 (92) |
| Age (years), median (IQR) | 13.4 (10.7, 14.9) | 12.4 (10.1, 15.0) |
| BMI z-score, median (IQR) | -0.73 (-1.67, 0.23) | -0.70 (-1.56, 0.29) |
| Disease Location |  |  |
| Colonic, n (%) | 25 (37) | 13 (35) |
| Ileal, n (%) | 6 (9) | 3 (8) |
| Ileocolonic, n (%) | 34 (52) | 21 (57) |
| upper GI/small bowel only, n (%) | 1 (2) | 0 (0) |
| Disease Behaviour |  |  |
| B1 (inflammatory), n (%) | 66 (100) | 37 (100) |
| B2 (stricturing), n (%) | 0 (0) |  |
| B3 (penetrating), n (%) | 0 (0) |  |
| Perianal involvement, n (%) | 6 (9) | 4 (11) |
| wPCDAI, median (IQR) | 42.5 (25, 60) | 35.0 (22, 57.5) |
| FCal (μg/g), median (IQR) | 1438 (1022, 1823) | 1610 (1136, 1865) |

^1^There were no statistically significant differences between patients enrolled in the main study and the group for which data are presented in the current study. Chi-square tests were used for categorical variables, and Mann-Whitney U tests for numerical variables. Data are presented with medians (Q1, Q3) or counts (%). GI: gastrointestinal tract, wPCDAI, weighted Paediatric Crohn’s Disease Activity Index, FCal, Faecal Calprotectin; BMI: Body mass index.

**Supplementary Table 2:** Pre-treatment stool characteristics, levels of targeted diet-related bacterial metabolites and microbial load, between responders and non-responders to treatment in children with Crohn’s disease who completed 8 weeks of EEN.

| Variables ^1^ | Responders N=13 | Non-Responders N=16 | P value |
| --- | --- | --- | --- |
| Short Chain Fatty Acids μmol/g (dry) |  |  |  |
| Acetate | 297 (250, 446) | 465 (288, 548) | 0.16 |
| Propionate | 72.6 (66.6, 99.1) | 90.9 (53.6, 136) | 0.50 |
| Butyrate | 64.9 (48.0, 86) | 112 (65.7, 162) | 0.04 |
| Isobutyrate | 8.04 (5.03, 12.6) | 11.5 (6.95, 15.7) | 0.33 |
| Isovalerate | 9.06 (5.79, 12.1) | 10.4 (8.26, 14.9) | 0.39 |
| Isocaproate | 0.93 (0.58, 2.16) | 1.31 (0.453, 2.25) | 0.84 |
| Valerate | 8.28 (1.44, 10.7) | 7 (1.63, 14.8) | 0.50 |
| Caproate | 0.78 (0.47, 1.68) | 0.735 (0.523, 1.51) | 0.91 |
| Heptanoate | 0.07 (0, 0.2) | 0.085 (0.023, 0.123) | 1.00 |
| Octanoate | 0.08 (0, 0.23) | 0.02 (0, 0.085) | 0.25 |
| Total SCFA | 482 (414, 614) | 674 (489, 877) | 0.10 |
| Short Chain Fatty Acids μmol/g (wet) |  |  |  |
| Acetate | 51.6 (45.8, 79.8) | 75.6 (60.4, 97.6) | 0.09 |
| Propionate | 12.3 (8.5, 18.2) | 16.9 (9.01, 30.1) | 0.50 |
| Butyrate | 13.0 (7.79, 16.9) | 20.6 (7.99, 30.5) | 0.06 |
| Isobutyrate | 2.01 (0.676, 3.59) | 2.29 (1.15, 3.33) | 0.71 |
| Isovalerate | 2.19 (0.78, 3.25) | 2.05 (1.31, 3.21) | 0.75 |
| Isocaproate | 0.193 (0.154, 0.287) | 0.208 (0.067, 0.444) | 0.98 |
| Valerate | 1.49 (0.15, 2.68) | 1.47 (0.334, 3.225) | 0.62 |
| Caproate | 0.145 (0.077, 0.39) | 0.137 (0.097, 0.296) | 0.88 |
| Heptanoate | 0.013 (0, 0.021) | 0.014 (0.003, 0.031) | 0.77 |
| Octanoate | 0.013 (0, 0.077) | 0.004 (0, 0.023) | 0.33 |
| Total SCFA | 82.0 (72.5, 109) | 133 (88.8, 165) | 0.06 |
| Hydrogen sulphide & ammonia |  |  |  |
| Total sulphide (per wet matter), nmol/g | 315 (121, 686) | 157 (106, 380) | 0.39 |
| Free sulphide (per wet matter), nmol/g | 14.4 (9.54, 25.5) | 9.42 (1.21, 26.9) | 0.40 |
| NH_3_ (per wet matter), mg/g | 2.9 (2.05, 3.35) | 3.40 (2.05, 5.00) | 0.57 |
| Faecal characteristics |  |  |  |
| Whole stool weight | 48.0 (20.9, 108) | 49.5 (19.2, 75.8) | 0.75 |
| %Water content | 81.3 (73.5, 86.4) | 81.4 (78.0, 84.6) | 0.87 |
| Bristol Stool scale | 6.0 (5.25, 7.0) | 6.0 (5.0, 7.0) | 0.83 |
| Faecal pH | 6.82 (6.40, 7.19) | 6.66 (6.17, 7.35) | 0.65 |
| Log_10_ gene copy number/g dry matter | 11.8 (11.7, 11.9) | 11.5 (10.7, 11.8) | 0.12 |

^1^ Numerical data are presented as median (IQR); EEN: Exclusive enteral nutrition; SCFA: Short chain fatty acids. EEN: Exclusive enteral nutrition. Mann-Whitney U tests were used for statistical analysis

**Supplementary Table 3:**  ^1^H NMR metabolites in faeces of responders and non-responders to treatment with EEN

| Metabolites (μg/g) ^1^ | Responders N=15 | Non-Responders N=19 | P value |
| --- | --- | --- | --- |
| 2-Hydroxy-3-methylvalerate | 1.92 (0.570, 2.65) | 2.47 (1.09, 3.74) | 0.238 |
| 3,4-Dihydroxyphenylacetic acid | 0.013 (0.013, 0.013) | 0.013 (0.013, 0.013) | NA |
| 3-(3-Hydroxyphenyl) propionic acid | 0.013 (0.013, 0.013) | 0.061 (0.013, 0.308) | 0.011 |
| 3-Hydroxyphenylacetate | 0.013 (0.013, 0.013) | 0.013 (0.013, 0.013) | NA |
| 4-Hydroxyphenylacetate | 0.013 (0.013, 0.013) | 0.013 (0.013, 0.108) | 0.561 |
| Acetate | 49.9 (46.3, 68.5) | 70.4 (57.0, 95.5) | 0.027 |
| Alanine | 7.70 (6.04, 11.1) | 9.32 (6.84, 16.0) | 0.256 |
| Butyrate | 13.1 (8.63, 18.4) | 22.3 (12.0, 31.9) | 0.030 |
| Choline | 0.12 (0.092, 0.296) | 0.161 (0.11, 0.363) | 0.425 |
| Glycine | 6.12 (3.41, 7.68) | 5.39 (3.79, 9.64) | 0.732 |
| Isobutyrate | 2.46 (0.68, 3.89) | 3.85 (1.81, 4.17) | 0.259 |
| Isoleucine | 3.21 (2.28, 4.55) | 3.87 (2.66, 5.49) | 0.410 |
| Isovalerate | 2.35 (0.83, 2.83) | 3.09 (1.81, 4.01) | 0.115 |
| Leucine | 4.20 (2.60, 5.47) | 4.83 (3.46, 8.47) | 0.157 |
| Lysine | 3.32 (2.12, 4.25) | 3.38 (2.09, 6.54) | 0.456 |
| Malonate | 0.714 (0.557, 0.927) | 0.625 (0.53, 1.01) | 0.656 |
| Methanol | 1.21 (0.722, 2.29) | 1.14 (0.982, 1.40) | 0.656 |
| Phenylacetate | 0.175 (0.013, 0.611) | 0.943 (0.438, 1.35) | 0.021 |
| Phenylalanine | 2.24 (1.67, 2.94) | 2.51 (1.48, 4.04) | 0.515 |
| Propionate | 16.5 (10.1, 19.8) | 20.9 (15.5, 30.4) | 0.051 |
| Putrescine | 0.013 (0.013, 0.882) | 0.798 (0.013, 2.135) | 0.192 |
| Succinate | 2.76 (0.619, 12.7) | 0.84 (0.538, 2.21) | 0.202 |
| Threonine | 2.37 (1.83, 2.97) | 2.72 (1.54, 4.55) | 0.451 |
| Tryptophan | 0.311 (0.218, 0.433) | 0.353 (0.214, 0.519) | 0.627 |
| Tyrosine | 1.83 (1.14, 2.00) | 1.92 (1.34, 3.20) | 0.202 |
| Uracil | 1.04 (0.628, 1.34) | 1.22 (0.630, 2.49) | 0.430 |
| Valerate | 1.91 (0.543, 3.88) | 3.10 (1.25, 4.99)  9) | 0.165 |
| Valine | 4.50 (3.48, 6.47) | 4.76 (3.17, 8.09) | 0.732 |
| p-Cresol | 0.079 (0.020, 0.374) | 0.130 (0.027, 0.245) | 0.889 |

^1^ Numerical data are presented as median (IQR); NA: non-applicable. EEN: Exclusive enteral nutrition; Mann-Whitney U tests were used for statistical analysis

**Supplementary Table 4:** Pre-treatment plasma cytokine concentrations of responders and non-responders to treatment in patients who completed 8 weeks of EEN

| Cytokines (pg/ml) ^1^ | Responders N=9 | Non-Responders N=21 | P value |
| --- | --- | --- | --- |
| TNF-α | 1.87 (1.31, 2.18) | 1.54 (1.25, 1.98) | 0.54 |
| IFN-γ | 20.8 (7.75, 52.9) | 30.0 (5.94, 49.3) | 0.71 |
| IL-1β | 0.113 (0.093, 0.187) | 0.127 (0.107, 0.201) | 0.41 |
| IL-2 | 0.422 (0.303, 0.603) | 0.312 (0.237, 0.376) | 0.19 |
| IL-4 | 0.026 (0.021, 0.034) | 0.026 (0.019, 0.033) | 0.85 |
| IL-6 | 2.21 (1.53, 3.97) | 2.03 (1.25, 3.84) | 0.90 |
| IL-8 | 6.78 (3.34, 13.0) | 6.63 (4.55, 10.5) | 0.65 |
| IL-10 | 0.249 (0.208, 0.433) | 0.269 (0.186, 0.368) | 0.87 |
| IL-12p70 | 0.144 (0.138, 0.162) | 0.198 (0.144, 0.263) | 0.16 |
| IL-13 | 2.88 (2.46, 4.01) | 4.01 (3.16, 6.19) | 0.09 |
| IL-17A | 9.75 (6.84, 16.6) | 10.6 (8.18, 14.8) | 0.90 |
| IL-17E | 5.00 (2.90, 11.2) | 3.98 (2.46, 5.24) | 0.10 |
| IL-17F | 1305 (577, 2312) | 1701 (897, 2042) | 0.93 |
| IL-21 | 979 (811, 1806) | 1456 (982, 1654) | 0.62 |
| IL-22 | 2.71 (1.58, 5.61) | 2.29 (1.89, 3.73) | 0.62 |
| IL-23 | 8.37 (4.45, 16.3) | 12.1 (6.50, 13.3) | 0.56 |
| IL-27 | 558 (341, 833) | 526 (424, 668) | 0.87 |
| IL-31 | 58.1 (37.4, 84.5) | 69.5 (48.6, 82.9) | 0.87 |
| IL-33 | 3.55 (1.69, 6.49) | 4.19 (2.48, 6.29) | 0.87 |

^1^ Numerical Data are presented as median (IQR). EEN: Exclusive enteral nutrition; Mann-Whitney U tests were used for statistical analysis

**Supplementary Table 5:** Normalised protein expression between responders and non-responders to treatment with EEN

| Protein ^1^ | Responders N=13 | Non-Responders N=18 | P value |
| --- | --- | --- | --- |
| NT.3 | 0.69 (0.69, 0.69) | 1.49 (0.69, 1.79) | 0.03 |
| CXCL6 | 11.1 (10.5, 11.2) | 10.5 (10.3, 10.9) | 0.05 |
| TRANCE | 4.07 (3.65, 4.8) | 5 (4.47, 5.44) | 0.06 |
| IL.18R1 | 8.65 (8.32, 8.82) | 8.14 (7.92, 8.52) | 0.06 |
| MMP.10 | 8.86 (8.56, 9.37) | 9.73 (8.81, 10.4) | 0.07 |
| AXIN1 | 2.1 (1.93, 3.13) | 2.67 (2.33, 2.93) | 0.09 |
| SIRT2 | 3.2 (2.99, 4.74) | 3.88 (3.31, 5.08) | 0.09 |
| IL8 | 6.24 (5.26, 7.25) | 6.76 (6.3, 7.45) | 0.10 |
| IL.15RA | 1.31 (1.15, 1.55) | 1.51 (1.4, 1.67) | 0.11 |
| TRAIL | 7.84 (7.63, 8.11) | 8.02 (7.9, 8.23) | 0.11 |
| CXCL5 | 11.9 (11.1, 12.6) | 11.4 (10.9, 11.8) | 0.11 |
| TNFB | 5.2 (5.04, 5.46) | 5.52 (5.23, 5.69) | 0.12 |
| IL4 | 0.49 (0.49, 0.49) | 0.49 (0.49, 1.47) | 0.15 |
| SLAMF1 | 1.01 (1.01, 1.01) | 1.01 (1.01, 1.83) | 0.19 |
| MCP.3 | 3.15 (3, 3.74) | 3.72 (3.16, 3.9) | 0.20 |
| HGF | 8.34 (8.13, 8.57) | 8.18 (7.91, 8.32) | 0.20 |
| LAP.TGF.beta.1 | 8.01 (7.51, 8.14) | 7.64 (7.45, 7.92) | 0.23 |
| NRTN | 0.66 (0.66, 0.66) | 0.66 (0.66, 0.66) | 0.24 |
| CD5 | 5.74 (5.34, 5.89) | 6.02 (5.49, 6.11) | 0.24 |
| SCF | 8.99 (8.32, 9.43) | 8.61 (8.13, 8.96) | 0.26 |
| CASP.8 | 2.45 (2.19, 2.83) | 2.66 (2.17, 3.12) | 0.26 |
| FGF.5 | 0.66 (0.66, 0.66) | 0.66 (0.66, 0.66) | 0.27 |
| Beta.NGF | 0.69 (0.69, 0.69) | 0.69 (0.69, 0.69) | 0.27 |
| IL33 | 0.89 (0.89, 0.89) | 0.89 (0.89, 0.89) | 0.27 |
| LIF | 0.54 (0.54, 0.54) | 0.54 (0.54, 0.54) | 0.27 |
| MCP.1 | 11.7 (11.3, 11.8) | 11.8 (11.5, 11.9) | 0.31 |
| CD40 | 11.2 (11.0, 11.5) | 11.3 (11.2, 11.6) | 0.31 |
| STAMBP | 4.12 (3.98, 5.1) | 4.49 (4.11, 5.67) | 0.31 |
| CCL28 | 0.86 (0.39, 1.09) | 1.03 (0.87, 1.27) | 0.32 |
| IL.10RB | 6.12 (5.97, 6.3) | 6.3 (5.95, 6.45) | 0.33 |
| EN.RAGE | 3.62 (3.25, 4.5) | 4.23 (3.56, 4.6) | 0.33 |
| GDNF | 0.97 (0.97, 0.97) | 0.97 (0.97, 0.97) | 0.35 |
| IL.22.RA1 | 1.1 (1.1, 1.1) | 1.1 (1.1, 1.1) | 0.36 |
| DNER | 9.12 (8.7, 9.43) | 8.82 (8.57, 9.04) | 0.42 |
| IL.20RA | 0.73 (0.73, 0.73) | 0.73 (0.73, 0.73) | 0.43 |
| IL.17A | 2.61 (2.36, 2.86) | 2.89 (2.19, 3.53) | 0.44 |
| IL6 | 4.41 (3.84, 5.17) | 4.17 (3.52, 4.47) | 0.46 |
| CST5 | 5.19 (5, 5.41) | 5.01 (4.85, 5.28) | 0.49 |
| Flt3L | 8.25 (7.85, 8.46) | 8.19 (7.82, 8.29) | 0.49 |
| X4E.BP1 | 7.31 (6.86, 8.43) | 8.03 (6.98, 9.24) | 0.49 |
| CXCL1 | 9.53 (8.8, 9.93) | 9.4 (9.04, 9.69) | 0.54 |
| MCP.2 | 8.31 (7.89, 8.87) | 8.68 (8.14, 8.87) | 0.54 |
| IL.10RA | 0.51 (0.51, 1.24) | 1.06 (0.51, 1.35) | 0.59 |
| CSF.1 | 10.1 (10.0, 10.3) | 10.0 (9.97, 10.3) | 0.59 |
| MMP.1 | 9.57 (8.75, 10.6) | 9.29 (8.92, 9.92) | 0.62 |
| IL.24 | 1.09 (1.09, 2.44) | 1.09 (1.09, 2.63) | 0.64 |
| IL7 | 2.79 (2.64, 3.11) | 2.7 (2.63, 3.04) | 0.65 |
| LIF.R | 4.06 (3.83, 4.27) | 4.06 (3.93, 4.25) | 0.65 |
| CCL20 | 7.68 (6.52, 8.11) | 7.57 (7.33, 8.07) | 0.65 |
| IL5 | 0.79 (0.79, 0.79) | 0.79 (0.79, 0.79) | 0.66 |
| FGF.21 | 4.68 (3.91, 5.39) | 4.72 (3.88, 6.76) | 0.69 |
| OPG | 9.36 (9.29, 9.62) | 9.51 (9.31, 9.63) | 0.74 |
| uPA | 10.1 (9.84, 10.4) | 10.2 (9.92, 10.3) | 0.74 |
| TNFSF14 | 4.33 (3.62, 4.54) | 3.95 (3.65, 4.68) | 0.74 |
| CCL19 | 9.1 (8.99, 9.99) | 9.37 (9.06, 9.86) | 0.74 |
| PD.L1 | 6.65 (6.45, 6.96) | 6.6 (6.43, 6.95) | 0.74 |
| IL.17C | 0.9 (0.9, 1.81) | 0.9 (0.9, 1.99) | 0.74 |
| ARTN | 0.36 (0.36, 0.36) | 0.36 (0.36, 0.36) | 0.75 |
| CD8A | 10.5 (10.3, 11.1) | 10.6 (10.3, 11.0) | 0.77 |
| CXCL11 | 13.0 (12.3, 13.5) | 13.1 (12.7, 13.3) | 0.77 |
| TGF.alpha | 3.79 (3.52, 4.01) | 3.79 (3.56, 3.99) | 0.77 |
| FGF.23 | 2.55 (2.36, 2.93) | 2.75 (2.44, 2.95) | 0.77 |
| IL.12B | 6.67 (6.36, 6.98) | 6.71 (6.43, 7.01) | 0.77 |
| TNF | 3.93 (3.52, 4.27) | 3.97 (3.7, 4.2) | 0.77 |
| CD244 | 7.17 (6.97, 7.34) | 7.16 (6.98, 7.43) | 0.80 |
| MCP.4 | 14.9 (14.7, 15.1) | 14.8 (14.5, 15.4) | 0.80 |
| CCL11 | 7.48 (7.26, 7.72) | 7.41 (7.3, 7.77) | 0.80 |
| IL10 | 3.85 (3.53, 4.83) | 3.91 (3.72, 4.32) | 0.80 |
| IFN.gamma | 8.57 (7.4, 10.11) | 8.9 (7, 9.93) | 0.80 |
| FGF.19 | 7.01 (6.87, 7.26) | 7.26 (6.7, 7.71) | 0.80 |
| ST1A1 | 3.39 (3, 3.55) | 3.37 (2.96, 3.79) | 0.80 |
| CCL4 | 6.46 (6.11, 6.66) | 6.27 (6.14, 6.62) | 0.83 |
| CCL3 | 5.41 (4.73, 5.68) | 5.19 (4.93, 5.51) | 0.83 |
| TNFRSF9 | 8.3 (8.04, 8.45) | 8.3 (7.95, 8.55) | 0.83 |
| ADA | 5.14 (4.99, 5.89) | 5.55 (5.14, 5.86) | 0.83 |
| CCL25 | 5.86 (5.41, 6.56) | 6 (5.63, 6.27) | 0.86 |
| CX3CL1 | 5.63 (5.55, 5.79) | 5.64 (5.45, 5.81) | 0.86 |
| IL.2RB | 0.85 (0.85, 0.85) | 0.85 (0.85, 0.85) | 0.86 |
| TSLP | 0.31 (0.31, 0.31) | 0.31 (0.31, 0.31) | 0.89 |
| CDCP1 | 2.98 (2.7, 3.59) | 2.97 (2.83, 3.26) | 0.89 |
| CD6 | 5.36 (5.32, 5.73) | 5.35 (4.96, 5.99) | 0.89 |
| VEGFA | 9.95 (9.72, 10.4) | 10.1 (9.71, 10.3) | 0.95 |
| CCL23 | 10.7 (10.3, 11.1) | 10.8 (10.3, 11.1) | 0.95 |
| OSM | 4.04 (3.57, 4.63) | 4.26 (3.25, 4.84) | 0.98 |
| CXCL9 | 9.6 (8.45, 10.6) | 9.72 (8.86, 10.2) | 0.98 |
| IL18 | 8.98 (8, 9.43) | 8.98 (8.56, 9.37) | 0.98 |
| CXCL10 | 10.3 (8.97, 10.5) | 10.0 (9.34, 10.5) | 1.00 |
| TWEAK | 9.56 (9.29, 9.62) | 9.48 (9.27, 9.65) | 1.00 |
| IL.1.alpha | 0.18 (0.18, 0.18) | 0.18 (0.18, 0.18) | NA |
| IL2 | 0.86 (0.86, 0.86) | 0.86 (0.86, 0.86) | NA |
| IL13 | 0.81 (0.81, 0.81) | 0.81 (0.81, 0.81) | NA |
| IL.20 | 0.55 (0.55, 0.55) | 0.55 (0.55, 0.55) | NA |

^1^ Numerical Data are presented as median (IQR); NA: non-applicable; EEN: exclusive enteral nutrition; Mann-Whitney U tests were used for statistical analysis
