# Supplementary material for: Gut metabolome and microbiota signatures predict response to treatment with exclusive enteral nutrition in a prospective study in children with active Crohn’s disease
Source: Am J Clin Nutr. 2024 Feb 19;119(4):885–95. doi: 10.1016/j.ajcnut.2023.12.027 (PMC11007740; doi:10.1016/j.ajcnut.2023.12.027)
Supplement: Supplementary file 4 [file mmc1.zip › mmc1.odt]

Ben Nichols. “Gut metabolome and microbiota signatures predict response to treatment with exclusive enteral nutrition in a prospective study in children with active Crohn’s disease”    

Supplementary Figure 1: Participant recruitment flowchart. There were 37 eligible participants who completed EEN and provided fecal samples. Of these, 15 responded to treatment (FCal < 250μg/g) and 22 did not (FCal > 250/kg). EEN: Exclusive enteral nutrition; wPCDAI: weighted paediatric Crohn’s disease activity index.

Supplementary Figure 2: Principal Component Analysis of 1H NMR metabolite concentration data for responders and non-responders. Ellipses for 95% standard deviation are shown. PCA: Principal Component Analysis; Non-RS: Fecal calprotectin non-responders; RS: Fecal calprotectin responders.

Supplementary Figure 3: Principal Component Analysis of inflammation-related plasma proteomics for responders versus non-responders. PCA: Principal Component Analysis; Non-RS: Fecal calprotectin non-responders; RS: Fecal calprotectin responders.

Supplementary Figure 4: Spearman correlation results for influential OTUs that correlated significantly with Acetate, Butyrate, Phenylacetate and 3-(3-hydroxyphenyl)propionic acid. OTU abundances have been transformed using total sum scaling normalization and centered log-ratio transformation and metabolite concentrations are μg/g. OTU: Operational taxonomic unit.
